# The Effect of Repeated Botulinum Toxin A Therapy Combined with Intensive Rehabilitation on Lower Limb Spasticity in Post-Stroke Patients

**DOI:** 10.3390/toxins10090349

**Published:** 2018-08-31

**Authors:** Takatoshi Hara, Masahiro Abo, Hiroyoshi Hara, Nobuyuki Sasaki, Naoki Yamada, Masachika Niimi, Yusuke Shimamoto

**Affiliations:** 1 Department of Rehabilitaion Medicine, The Jikei University School of Medicine, 1058461 Tokyo, Japan; abo@jikei.ac.jp (M.A.); nobsasa1005@gmail.com (N.S.); mela012921@yahoo.co.jp (N.Y.); pomardon2010@gmail.com (M.N.); 2 Department of Rehabilitaion Medicine, Kikyogahara Hospital 1295, 3996461 Nagano, Japan; hhara448@orange.plala.or.jp (H.H.); shimamoto@keijin-kai.jp (Y.S.)

**Keywords:** botulinum toxin A therapy, stroke, rehabilitation

## Abstract

Objectives: This study is a retrospective investigation of the effects of repetitive botulinum toxin A therapy (BoNT-A) and intensive rehabilitation (IR) on lower limb spasticity in post-stroke patients. Methods: Thirty-five post-stroke patients was included in this study and received BoNT-A for the first time. A 12-day inpatient protocol was with 4 cycles of the treatment protocol. The severity of spasticity, motor function and brace status were evaluated. Results: The modified Ashworth Scale (MAS) score of ankle dorsiflexors, range of motion, walking speed and balancing ability were significantly improved after cycle 1. The improvement of spasticity and motor function was persistent through cycles 2–4. One-third of brace users were able to discontinue the use of a brace. All of these brace users showed a forward gait pattern prior to therapy. Conclusions: Repeated BoNT-A combined with IR improved lower limb spasticity in post-stroke patients. Our results suggest that patients who show the forward gait pattern prior to therapy may be able to discontinue the use of their brace after therapy.

## 1. Introduction

Botulinum toxins are produced by *Clostridium botulinum* bacteria and are the etiological agents of botulism, a rare but severe disease for animals [1,2]. But, for their biological and toxicological features, Botulinum toxins have become sophisticated tools to study neuronal physiology and valuable therapeutics for an increasing number of human disorders [2]. Botulinum toxins have been classified into seven different serotypes (from BoNT/A to /G). Type A have been clinically used for spasticity [3]. Botulinum toxin A therapy (BoNT-A) temporarily reduce muscle activity by preventing the release of acetylcholine at the neuromuscular injection, resulting in reduced spasticity and muscle tone [4]. The effect of intramuscular injection of botulinum toxin type A pharmacologically commence 2–4 days following injection, with the expected peak effect at 3 weeks [5]. Several open and placebo controlled studies have reported the efficacy of local botulinum toxin injections in reducing spasticity and empathizing its easy use and safety [6,7,8,9,10,11].

Post-stroke patients with hemiparesis may present with spasticity, a symptom of upper motor neuron syndrome [12]. Spasticity is observed in 19% of patients at 3 months after stroke and 38% of patients at 12 months after stroke [13,14]. Spasticity can interfere with rehabilitation and the muscle atrophy due to on-going spasticity, as well as the joint contractures and pain due to shortening of muscle fibers and ligaments can prevent improvement of activities of daily living (ADL) and patient reintegration into society [6]. Furthermore, lower limb spasticity limits the range of motion (ROM) of the joint and the adjustment of muscle tone that are required for successful walking [7]. Lower limb spasticity can result in the sustained over activity of the triceps surae muscle, which then leads to equinus of the foot [15]. The equinus foot can cause ankle instability during the loading response phase and poor toe clearance during the swing phase of gait [16]. Previous randomized controlled trials (RCTs) indicated that BoNT-A reduces spasticity [8,9]. With regard to the lower limb, BoNT-A injections have been reported to be effective for the improvement of pes varus and equinovarus foot, reduction of clonus and improvement of walking velocity [15]. A systematic review of gait velocity in RCTs reported a 0.044 m/s increase (an effect size of 0.193) in gait velocity in the treatment groups, although the number of studies reporting such an improvement was small [17]. Therefore, more recent studies have attempted the combined use of BoNT-A and rehabilitation to improve the motor function of patients.

Previously, our study group implemented a combined treatment program of BoNT-A therapy with multidisciplinary rehabilitation (MD-Re) and reported that this combined treatment was effective for the improvement of motor function in post-stroke patients with upper and lower limb spasticity [18]. In addition, the effects of the combined treatment program varied depending on the extent of changes in muscle fibers induced by sustained spasticity. Improvement of motor function was observed in a case where muscle echo intensity was low but the improvement was poor in a case where muscle echo intensity was high [19]. It has been known that the efficacy of BoNT-A attenuates in 3–4 months after administration [6,7]. Therefore, the suppression of the development of persistent spasticity may contribute to the maintenance and improvement of motor function in the long-term.

A previous study using the repeated administration of BoNT-A provided firm evidence for the efficacy and safety of BoNT-A [10]. In addition, studies on post-stroke spasticity reported the persistent effect of repeated administration of BoNT-A on spasticity and maintenance of patients’ functions [11,20,21]. Only a few studies, however, have reported BoNT-A therapy for lower limb spasticity and reports on the combined treatment with BoNT-A therapy and rehabilitation are further limited [22].

Therefore, we examined retrospectively the effect of the combined therapy of repeated BoNT-A administration and rehabilitation to clarify whether the combined treatment improved spasticity, whether the effect was maintained and whether it improved and maintained the motor function of the lower limbs.

## 2. Results

### 2.1. Study Population Characteristic and Transition of BoNT-A Therapy

The study subjects included 35 patients and excluded 1 patient with post cerebral traumatic spasticity. All patients completed 4 cycles of the inpatient treatment protocol. No patient postponed the treatment due to the reduction of spasticity. No patient dropped out of or deviated from the treatment cycle. Table 1 shows the characteristics of the patients in the current study. The patterns of gait were classified into two types according to the relative positions between the affected and unaffected feet in stepping the unaffected heel on the floor; forward or even. Table 2 shows the relationship between the pattern of gait and the type of brace. The Gait Solution Design (Kawamura Gishi Co., Ltd, Osaka, Japan) had the largest number of users [23,24]. The sites of injection and the dosage are summarized in Table 3. In regard to the interval of injection, Cycle1-2, Cycle2-3 and Cycle3-4 were 18.1 ± 3.57 weeks, 16.2 ± 3.69 weeks and 15.7 ± 3.66 weeks, respectively and no significant difference was observed.

### 2.2. Effect of Repeated BoNT-A Therapy Combined with Intensive Rehabilitation

Table 4 shows the changes in the assessment parameters in the 4 inpatient treatment cycles compared with the parameters before the intervention. Regarding the upper limbs, MAS scores for the shoulder, elbow, wrist and finger improved after Cycle 1 (Cycle 1 post) compared to the baseline values and the improvements persisted until after Cycle 4 (Cycle 4 post) (*p* < 0.05). Similarly, FMA scores also improved compared to the baseline values and the improvements persisted until after Cycle 4 (Cycle 4 post) (*p* < 0.05). As for the lower limbs, the MAS score for ankle dorsiflexors was significantly improved after Cycle 1 (Cycle 1 post) and the improvement was persistent until after Cycle 4 (Cycle 4 post) (*p* < 0.05). Similar improvements and their persistence were observed in the range of motion (ROM) at ankle dorsiflexion, 10-meter walking time (10MWT), functional reach test (FRT) scores and the result of the timed up and go test (TUG) (*p* < 0.05).

**Figure 1:**
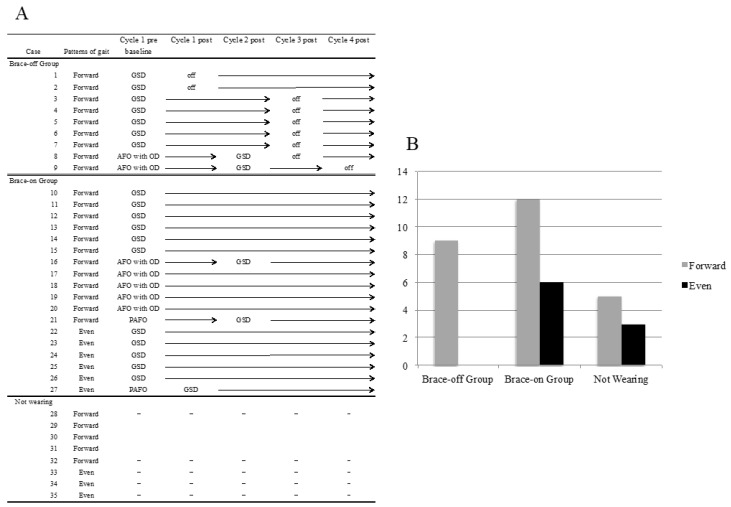
Brace changes and the relationship between pattern of gait and with or without brace at discharge. (**A**). The Change of brace. AFO with OD, Ankle-foot orthosis with an oil damper; GSD, Gait Solution Design; PAFO, Plastic ankle-foot orthosis. (**B**). The relationship between pattern of gait and with or without brace at discharge in Cycle 4. The vertical axis indicates the number of patients.

Figure 1A shows the change in the use status of braces in patients. Some patients were able to change their brace due to the improvement of spasticity and ROM of the ankle. In addition, one-third of patients who initially wore a brace no longer needed the brace at the end of Cycle 4 (Cycle 4 post). Regarding the relationship between gait pattern of the patients and their use status of braces at the end of Cycle 4 (Cycle 4 post), the gait pattern of all of patients who came off of a brace (Brace-off group) was the forward gait pattern (Figure 1B).

### 2.3. Comparison between Three Groups

**Figure 2:**
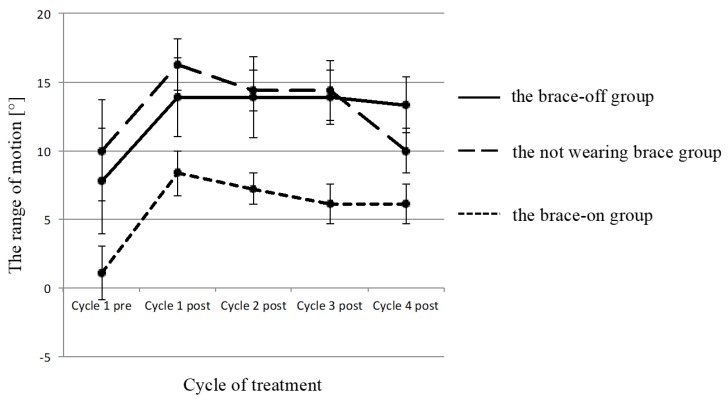
A comparison of the ROM of the ankle between the three groups. Dotted line, solid line and broken line indicate the brace-on group, the brace-off group and the not wearing brace group, respectively. ROM of the ankle was significantly larger in the not wearing brace group and the brace-off group than in the brace-on group from post cycle 1 to post cycle 4.

The patients were classified into the following three groups: patients who did not wear a brace throughout the study (Not wearing group); those who came off of a brace (Brace-off group); and those who continued to use a brace (Brace-on group). The ROM of the ankle was significantly better in the not wearing group and the brace off group than in the brace on group (*p* > 0.05) (Figure 2) but no significant differences were observed in other parameters among the groups.

## 3. Discussion

In the present study, we implemented a combined treatment program of repeated BoNT-A therapy and rehabilitation for post-stroke patients with spasticity and examined its effect on spasticity, motor function and the use of braces. Our results demonstrated an improvement of spasticity by the combined treatment program and maintenance of the improvement. In addition, the combined treatment enabled improved lower limb motor functions in the post-stroke patients and their brace use status. This is the first report on the combined treatment program of repeated BoNT-A therapy and intensive rehabilitation.

In this study, there was the high standard deviation in the age at injection and time between onset and treatments. Several years have passed since BoNT-A therapy in Japan. Therefore, BoNT-A therapy has become the available tool for spasticity in post-stroke patients. Thus, it was difficult to find patients who had not been treated with BoNT-A therapy. And, compared with the subject date of clinical trials in Japan, the average scores of the age at injection and time between onset and treatments were similar [6,7]. In this study, the youngest patient was the 30s and the oldest patient was the 80s in years of age. In the time between onset and treatments, one patient was 488 months and apart from this patient, three patients were over 100 months. Therefore, it is thought that the number of cases affected the standard deviation. There were more patients with a forward gait pattern than patients with an even gait pattern. In addition, there were more patients who used a brace than patients who did not use a brace prior to the intervention. Ankle-foot orthotic therapy for patients with post-stroke paresis and spasticity not only prevents contracture and deformity of joints and mitigates spasticity but also plays a pivotal role in regaining walking ability and relearning motor functions [25]. An ankle-foot orthosis with an oil damper and Gait Solution Design is especially effective to promote the forward driving force in the gait cycle by assisting heel rocker function [24]. The patients included in the current study that used ankle-foot orthotic devices prior to the intervention were most likely to benefit from these effects and we speculate that many of these patients attained the forward gait pattern as a result. A previous study demonstrated that the combined use of BoNT-A injection and orthotic therapy improved walking speed, increased peak ankle dorsiflexion during the stance phase and increased the peak ankle plantarflexion moment during the swing phase [26]. The combined use of an orthosis with BoNT-A therapy may have a substantial influence on the improvement of walking ability.

As for the changes in the total dose of BoNT-A and its injection site, the total dose of BoNT-A in a cycle and the interval between cycles (weeks) did not change significantly. The most likely reason for no significant changes in the interval between cycles was because the interval was set between 12 and 20 weeks in the treatment protocol. The total dose of BoNT-A in each cycle and its distribution into individual muscles were decided at the time of medical examination for each cycle. We speculated that the combined treatment with BoNT-A injection and intensive rehabilitation would reduce the total dose of BoNT-A gradually through repeated treatment but the total dose remained rather constant. On the other hand, with regard to the mean injected dosage of BoNT-A into individual muscles, the distributions of BoNT-A into the tibialis posterior muscle, gastrocnemius muscle and soleus muscle were constant, while the distribution into the tibialis anterior muscle decreased and the distributions into the flexor hallucis longus muscle and flexor digitorum longus muscle increased during the current study. This finding suggests that gradual improvement of varus and equinus conditions with repeated treatment made it possible for the dose of BoNT to be distributed into other muscles. In the future, it will be necessary to observe the long-term changes in the total dose and distribution to individual muscles over a treatment course.

We demonstrated a significant reduction of MAS scores in the upper and lower limbs and the maintained this reduction during the study period in the current treatment protocol. These results are consistent with those in previous studies [11,20,21]. In the upper limb, improvement of upper limb function, as well as the reduction of MAS scores were observed. A previous study demonstrated that repeated treatment with BoNT-A improved upper limb function, as well as spasticity in patients with post-stroke upper limb spasticity [27]. In addition, in relation to the improvement of lower limb function that will be discussed below, BoNT-A therapy on the upper limb may improve gait disturbance since by improving balancing ability and walking speed [28,29]. The mitigation of upper limb spasticity improves walking ability through the improvement of the position, swinging ability and voluntary movement of the upper limb. In the lower limb, the combined treatment of BoNT-A therapy and intensive rehabilitation not only improved MAS scores and the ROM of the ankle but also improved motor function and balancing ability. These effects of the combined treatment persisted throughout the study. A previous study reported an improvement of the MAS score of the ankle and walking ability by the repeated administration of BoNT-A [22] and our present results supported those findings. We speculate that the combined use of intensive rehabilitation and BoNT-A therapy creates a favorable environment to improve motor function and spasticity through listening to the patient’s needs at every treatment administration. Therefore, the combination therapy allowed adjustment of the orthotic device or to have the patient stop using the device. Teasell et al. examined the rehabilitation management of post-stroke patients and reported that proper management and training for spasticity was effective for the improvement of motor function in post-stroke patients, even at 6 months or more after onset [30]. The results of the present study support their findings.

All of the patients in the brace-off group showed the forward gait pattern. With regard to the mechanism of improvement, the reconstruction of neuroplasticity and the relearning of motor functions, the mechanism for acquisition of a normal pattern of motor function is very important. Therapeutic intervention to induce patients’ relearning of such motor functions is also important. Therefore, our results suggest that the combined treatment program contributed to the relearning of motor functions. In addition, our results suggest the importance of leading patients to the forward gait pattern in the post-stroke acute or subacute phase prior to the introduction of the treatment for spasticity. Furthermore, it is important to examine the types of patients that can acquire the forward gait pattern based on the type of stroke and the size of cerebral damage. A recent study examined the therapeutic effect of BoNT-A for spasticity in the early phase after the onset of stroke and demonstrated a significant reduction of MAS scores for the ankle [31]. Relearning and re-establishing walking ability and balancing ability is important for rehabilitation in the early phase after the onset of stroke. Therefore, the significance of a combined treatment using BoNT-A therapy and rehabilitation is clear in regard to the suppression of spasticity and the learning of a proper gait pattern.

Lastly, there are several limitations to the present study. First, this was a retrospective study with no control group. Ideally, we should have set an intensive rehabilitation only group or a BoNT-A monotherapy group. Second, the number of cases included in the current study is small. Several years have passed since BoNT-A therapy for post-stroke patients became widely available in Japan, thus, it was difficult to find patients who had not been treated with BoNT-A therapy. Third, it is necessary to follow patients who continued the use of a brace after the 4 cycles of treatment. In this study, the patients were followed for 12 or 15 months with 4 cycles of treatment but it may be necessary to observe their clinical course and the persistent effect of the treatment. In addition, there is a possibility that the patients who no longer required a brace may require a brace in the future if their walking ability decreases. Therefore, we will conduct a long-term follow-up of the patients on their clinical course and continue the treatment in the future.

## 4. Conclusions

The combined treatment of repeated BoNT-A therapy and intensive rehabilitation for lower limb spasticity in post-stroke patients mitigated spasticity and the effect was persistent throughout the study. Our results indicate that the combined treatment is effective for the improvement of lower limb motor function and the maintenance of the improvement. In addition, our results suggest that patients who showed the forward gait pattern before starting the intervention may be able to stop using a brace during or after treatment.

## 5. Materials and Methods

### 5.1. Study Design

**Figure 3:**
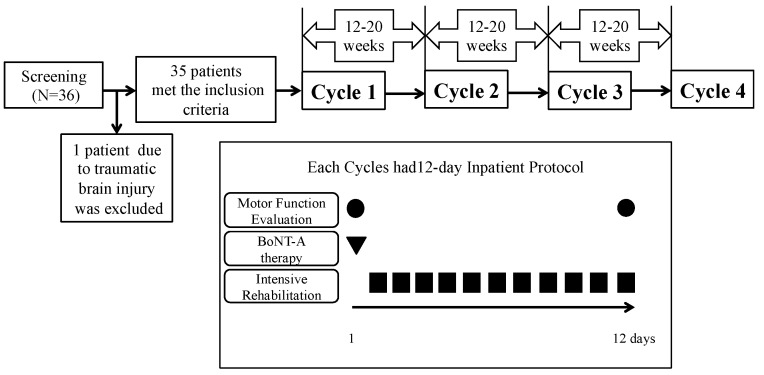
The study design. The study subjects included 35 patients and excluded 1 patient with. post cerebral traumatic spasticity. 35 patients completed 4 cycles of the inpatient treatment protocol. One treatment cycle was defined as a 12-day inpatient treatment protocol. At admission, all patients’ motor function before BoNT-A injections was evaluated. Beginning on the following day, the rehabilitation program was administered for 11 consecutive days. At the last day of this protocol, the motor function after intensive rehabilitation was evaluated for all patients. As for the interval between BoNT-A injections, BoNT-A was injected again between 12 weeks to 20 weeks after the last injection. Abbreviation: BoNT-A, botulinum toxin type A.

Figure 3 shows a flow chart of the study. Study subjects were patients introduced to BoNT-A therapy for the first time during a period from April 2014 through January 2017. One treatment cycle was defined as a 12-day inpatient treatment protocol including BoNT-A therapy, intensive rehabilitation and assessment of motor function before and after the hospitalization. As for the interval between BoNT-A injections, BoNT-A was injected again between 12 weeks to 20 weeks after the last injection. The patients underwent 4 cycles of the inpatient treatment protocol. If the modified Ashworth scale (MAS) score was 1 or below, the treatment was postponed.

### 5.2. Participants

We conducted an investigation of patients who visited Kikyogahara Hospital for BoNT-A injections and inpatient rehabilitation. The following inclusion criteria were used: (1) patients with hemiplegia following a stroke that involved upper and lower limb spasticity, (2) MAS of the ankle score ≥2 at cycle 1, (3) >6 months since the onset of stroke, (4) no prior BoNT-A injections, (5) no contraindications for BoNT-A injections and (6) the patient desire of additional improvements of hemiplegia. The exclusion criteria were: (1) only an upper limb being appropriate for BoNT-A injections, (2) unable to walk without assistance, (3) taking anti-spasticity medications, (4) a history of BoNT-A injections, (5) a history of surgery for spasticity due to stroke and (6) severe cognitive impairment. 

The current study was compliant with the Declaration of Helsinki and written informed consent was obtained from all participants. The current study was conducted following approval from the institutional ethics committee of Jikei University School of Medicine and Kikyougahara Hospital. This study was registered with the University Hospital Medical Information Network Clinical Trials Registry (UMIN-CTR) (UMIN 000033463).

### 5.3. Procedures

BoNT-A was administered based on the guidelines of Sheean et al. [32]. The maximum doses for the upper limb, lower limb and total were 240 U, 300 U and 360 U, respectively. BoNT-A (OnabotulinumtoxinA) was diluted with saline to a concentration of 25 U/mL. A team consisting of two physicians, an occupational therapist and a nurse observed the degree of upper and lower limb paresis, the degree of muscle contraction, the extent of dysfunction due to paresis and spasticity and the affected ADL. On the basis of these observations, the team planned the sites and dosage of BoNT-A injection by estimating the possibility of upper and lower limb functional improvement due to the reduction of muscle contraction. All patients were injected by the same physician. Ultrasonography was used to guide BoNT-A injections for all muscles. As the effects of BoNT-A are dose-dependent, we determined the dosages according to previous studies, the degree of spasticity and clinical experience, within the range of maximum dosages mentioned above [7,8,9].

### 5.4. Motor Function Evaluation

With regard to the MAS, we assessed the shoulder, elbow, wrist, finger, knee and ankle. The MAS was shown to have high reliability as a tool for measuring spasticity [33]. The degree of spasticity is classified into a total of 6 stages: 0, no increase in muscle tone; 1, Slightly increase in muscle tone, manifested by a catch and release or by minimal resistance at the end of the range of motion when the affected parts is moved in flexion or extension; 1+, Slight increase in muscle tone, manifested by a catch, followed by minimal resistance throughout the remainder (less than half) of the ROM; 2, More marked increase in muscle tone through most of the ROM but affected parts easily moved; 3, Considerable increase in muscle tone, passive movement difficult; 4, Affected parts rigid in flexion or extension. ROM measurements were obtained for hip flexion, knee extension and ankle dorsiflexion (the patient was resting supine on the examination table with their knees extended). ROM is presented the angular of the joint and is expressed in degree. In post-stroke patient, the limitation of ROM clinically is caused by spasticity due to a symptom of upper motor neuron syndrome. The Fugl-Meyer Assessment (FMA) is a comprehensive assessment battery of motor function that includes assessment of motor function of the upper and lower limbs [34]. In the upper limb, we used 33 items (maximum points of 66) of the FMA pertaining to upper limb function, including shoulder, elbow, forearm, wrist and hand. Each item is rated on a three-point ordinal scale: 0, cannot; 1, can perform partially; 2, can perform fully. Comfortable gait velocities of the 10 Meter Walk Test (10MWT) were also measured. The patients walked for 10 m in a straight line, with a 2-m run-up. Time was measured with a stopwatch and recorded in seconds. The Functional Reach Test (FRT) is used primarily to evaluate the patient fall risk [35] and reflects static balance [36]. This functional reach is defined as the maximal distance that one can reach forward beyond arm’s length, while maintaining a fixed base of support in the standing position.

Clinically, it is considered that FRT values less than 15 cm indicate a high risk of falls. We conducted this assessment to monitor changes in balance ability due to BoNT-A injections to both the upper and lower limbs. The Timed Up and Go (TUG) test is associated closely with static and dynamic balance, walking ability and ADL. It is an easy-to-administer test, yet, it has been shown to have high reliability [37,38]. In this test, the patients were required to stand up from a chair with armrests, walk 3 m, turn around, return to the chair and sit down as quickly as possible. Similar to the 10MWT, the time to completion was measured in seconds with a stopwatch. The patients completed 3 trials. 

In addition, the use status of a brace, type of the brace and the gait pattern were surveyed during the protocol treatment. Change in the brace used were performed as necessary in the rehabilitation program described below.

### 5.5. Rehabilitation Programs

All patients received BoNT-A injections in the afternoon of the day of admission. Beginning on the following day, the rehabilitation training was administered for 11 consecutive days. Including the assessment on the day of discharge, the rehabilitation program was a 12-day inpatient protocol. For the rehabilitation program, we implemented MD-Re. In MD-Re, a team of specialists was formed consisting of two physicians, a physical therapist, an occupational therapist and nurses. These members supported the patient and created a rehabilitation program to be shared between the patient and practitioners, based on the results of the assessment at admission. The team listened to the patient’s wishes and needs in creating a rehabilitation plan. The rehabilitation goals of each patient were different, therefore the rehabilitation approach also differed for each patient. Each patient received rehabilitation personally with a therapist. All patients participated in an individually tailored goal-oriented rehabilitation program that was developed based on their real-life demands. As the rehabilitation program aimed to improve not only spasticity but also patient functioning, ADL training, walking training, balance training, core training and brace adjustment or brace-off training were included, as well as stretching, positioning and ROM training. Outpatient rehabilitation was not implemented and the patients were instructed to continue the training regimen provided on the last day of the self-rehabilitation program at home after discharge.

### 5.6. Statistical Analyses

Data obtained at hospital discharge of each treatment cycle were compared with those obtained at the baseline. For the MAS, we converted scores of 1+ into 1.5 upon analysis. Normality was examined using the Shapiro-Wilk test. The data were analyzed by an analysis of variance with repeated measurements if they were normally distributed and data were analyzed by Friedman test if they were not normally distributed. In addition, patients were classified into three groups (“not wearing brace” group, “brace-off” group and “brace-on” group) according to their status of the use of a brace. These data were analyzed comparatively among the groups. 

Bonferroni corrections were used for multiple comparisons. All analyses were performed using SPSS 21.0 software (IBM, Armonk, NY, USA). A *p*-value < 0.05 indicated statistical significance in all tests. 

## Figures and Tables

**Table 1:** Subject data.

	All Patients
	(*n* = 35)
Age at injection, years (SD)	60.6 (11.1)
Male / Female, *n* (%)	27 (77.1)/8 (22.9)
Type of stroke, *n* (%)	
Cerebral infarction	13 (37.1)
Intracerebral hemorrhage	22 (62.9)
Side of hemiparesis	
Rt/Lt, *n* (%)	17 (48.5)/18 (51.5)
Brunnstrom recovery stage, median (IQR)	
Lower limb	4 (1)
Time between onset and treatment, months (SD)	47.0 (92.8)
Patterns of gait, *n* (%)	
Forward: Wearing Brace/Not wearing	21 (80.7)/9 (19.3)
Even Wearing Brace/Not wearing	6 (66.6)/3 (33.4)

SD, standard deviation; Rt, right; Lt, left; IQR, interquartile range.

**Table 2:** The relationship between the pattern of gait and the type of brace.

Type of Brace	Forward	Even
	*n* = 21 (%)	*n* = 6 (%)
Plastic ankle-foot orthosis	1 (4.8)	1 (16.6)
Ankle-foot orthosis with an oil damper	7 (33.3)	0
Gait Solution Design	13 (61.9)	5 (83.4)

**Table 3:** Mean average injected dosage of botulinum toxin type A and the frequency of botulinum toxin type A injection for each muscle.

Muscle, SD (%)	Cycle 1	Cycle 2	Cycle 3	Cycle 4	*p*-Value
Total Upper Limb	148.2 ± 66.2	154 ± 62.6	132.9 ± 59.5	148.8 ± 59.5	n.s.
Subscapularis	1.57 ± 6.50 (5.7)	5.14 ± 10.4 (20.0)	11.57 ± 14.1 (42.8)	11.57 ± 14.1 (42.8)	
Greater pectoral	12.7 ± 13.9 (45.7)	11.4 ± 16.4 (40.0)	8.71 ± 14.9 (28.5)	10.0 ± 16.2 (31.4)	
Teres major	2.14 ± 7.10 (8.5)	3.57 ± 8.87 (14.2)	10.5 ± 14.5 (37.1)	9.28 ± 13.4 (34.2)	
Biceps	26.5 ± 21.8 (68.5)	27.1 ± 18.5 (77.2)	19.4 ± 19.5 (57.1)	30.4 ± 27.4 (77.2)	
Brachioradialis	2.85 ± 8.07 (11.4)	2.14 ± 7.10 (8.5)	0 (0)	0.7 ± 4.22 (2.8)	
Flexor carpi radialis	19.4 ± 16.2 (65.8)	23.7 ± 14.8 (80.0)	16.2±18.8 (51.4)	22.6 ± 20.1 (65.0)	
Flexor carpi ulnaris	13.0 ± 14.2 (48.5)	12.7 ± 12.5 (51.4)	11.4 ± 15.2 (40.0)	5.8 ± 10.9 (22.8)	
Flexor digitorum superficialis	36.1 ± 23.0 (85.7)	35.0 ± 18.9 (85.7)	28.1 ± 21.6 (71.4)	32.4 ± 21.6 (80.0)	
Flexor digitorum profundus	16.0 ± 17.5 (54.2)	14.4 ± 14.0 (54.2)	19.3 ± 17.7 (62.8)	14.6 ± 14.6 (54.2)	
Flexor pollicis longus	9.21 ± 12.5 (37.1)	8.21 ± 11.7 (34.2)	1.78 ± 6.17 (8.5)	7.14 ± 11.4 (28.5)	
Adductor muscle of thumb	2.0 ± 6.66 (8.5)	2.92 ± 7.58 (14.2)	2.14 ± 7.10 (8.5)	1.42 ± 5.88 (5.7)	
Lumbricales	2.35 ± 6.88 (11.4)	3.28 ± 8.30 (14.2)	0 (0)	2.14 ± 7.10 (8.5)	
Total Lower limb	197 ± 55.4	192.5 ± 59.4	221 ± 55.7	205.4 ± 57.4	n.s.
Hamstring	6.42 ± 14.0 (20.0)	8.71 ± 20.2 (22.8)	11.5 ± 20.5 (28.5)	8.71 ± 14.9 (28.5)	
Rectus femoris	9.0 ± 20.5 (20.0)	15.7 ± 25.0 (34.2)	20.4 ± 25.2 (45.7)	14.1 ± 24.1 (31.4)	
Tibialis anterior	26.0 ± 23.5(74.3)	17.4 ± 17.0(60.0)	17.3 ± 20.4(54.2)	11.7 ± 17.7(34.2)	
Tibialis posterior	37.5 ± 21.7(94.1)	27.4 ± 15.7(82.8)	36.4 ± 23.3(85.7)	37.6 ± 22.3(82.8)	
Flexor hallucis longus	6.85 ± 11.1 (28.5)	13.8 ± 16.2 (45.7)	21.0 ± 19.9 (62.8)	19.5 ± 17.3 (62.8)	
Flexor digitorum longus	4.71 ± 10.7 (17.1)	8.71 ± 12.4 (34.2)	12.1 ± 15.3 (42.8)	9.28 ± 14.9 (31.4)	
Gastrocnemius	62.0 ± 30.1 (97.1)	60.0 ± 28.4 (97.1)	59.7 ± 24.9 (94.2)	65.2 ± 30.9 (94.2)	
Soleus	37.7 ± 18.9 (91.4)	36.4 ± 15.6 (94.2)	35.1 ± 25.4 (77.1)	34.1 ± 24.8 (80.0)	
Total	344.7 ± 26.3	346.5 ± 34.8	352.4 ± 19.5	351.4 ± 21.5	n.s.

SD, standard deviation. % indicates the injection frequency of patients in each group.

**Table 4:** Change in assessment values.

Assessment	Cycle 1 Pre	Cycle 1 Post	Cycle 2 Post	Cycle 3 Post	Cycle 4 Post
	Baseline				
Brunnstrom recovery stage, median (IQR)					
Upper limb	3 (1)	3 (1)	3 (1)	3 (1)	3 (1)
Finger	3 (1)	3 (1)	3 (1)	3 (1)	3 (1)
Lower limb	4 (1)	4 (1)	4 (1)	4 (1)	4 (1)
MAS, median (IQR)					
Shoulder flexors	1.5 (1)	1 (0.5) *	1 (0.5) *	1 (0.5) *	1 (0) *
Elbow flexors	2 (0.5)	1.5 (0.5) *	1 (0.5) *	1.5 (0.5) *	1.5 (0.5) *
Wrist flexors	2 (0.5)	1 (0.5) *	1 (0.5) *	1 (0.5) *	1 (0.25) *
Finger flexors	2 (0.5)	1 (0.5) *	1 (0.5) *	1 (0.5) *	1 (0.5) *
Knee extension	1 (1.5)	1 (1)	1 (1)	1 (1)	1 (1)
Ankle dorsiflexors	2 (0.5)	1.5 (0.5) *	1.5 (0.5) *	1 (0.5) *	1 (0.5) *
ROM, angle, average (SD)					
Hip flexion	109.6 (9.98)	111 (8.95)	109.6 (9.75)	110.6 (9.44)	112.1 (8.54)
Knee extension	132.5 (10.5)	135.1 (7.12)	130.0 (23.9)	133.8 (7.48)	133.4 (6.83)
Ankle dorsiflexion	4.85 (10.1)	11.5 (7.74) *	10.6 (6.83) *	10.0 (7.17) *	8.85 (6.42) *
FMA, Upper limb median (IQR)	14 (17)	16 (18) *	17 (18.5) *	19 (20) *	19 (18.5) *
10MWT, s, median (IQR)	26.9 (31.9)	23.2 (21.6) *	18.7 (22.4) *	19.8 (23.5) *	18.9 (17.1) *
FRT, cm, median (IQR)	15.5 (10.6)	22.5 (10.8) *	22.0 (7.0) *	22.0 (6.5) *	25.0 (11.0) *
TUG, s, median (IQR)	27.0 (31.5)	22.3 (21.9) *	22.1 (17.9) *	21.1 (21.7) *	20.2 (19.7) *

* Statistically significant difference between admission and discharge (*p* < 0.05). IQR, interquartile range. SD, standard deviation. FMA, Fugl-Meyer Assessment.
